# Sweepstake evolution revealed by population-genetic analysis of copy-number alterations in single genomes of breast cancer

**DOI:** 10.1098/rsos.171060

**Published:** 2017-09-27

**Authors:** Mamoru Kato, Daniel A. Vasco, Ryuichi Sugino, Daichi Narushima, Alexander Krasnitz

**Affiliations:** 1 Department of Bioinformatics, National Cancer Center Research Institute, 5-1-1 Tsukiji, Chuuoo-ku, Tokyo 104-0045, Japan; 2 Department of Bioengineering and Therapeutic Sciences, University of California, San Francisco, CA 94158, USA; 3 School of Advanced Sciences, The Graduate University for Advanced Studies, Hayama, Kanagawa 240-0193, Japan; 4 Cold Spring Harbor Laboratory, Simons Center for Quantitative Biology, One Bungtown Road, Cold Spring Harbor, NY 11724, USA

**Keywords:** bioinformatics, cancer genomics, copy-number alteration, molecular evolution, single-cell sequencing, coalescent theory

## Abstract

Single-cell sequencing is a promising technology that can address cancer cell evolution by identifying genetic alterations in individual cells. In a recent study, genome-wide DNA copy numbers of single cells were accurately quantified by single-cell sequencing in breast cancers. Phylogenetic-tree analysis revealed genetically distinct populations, each consisting of homogeneous cells. Bioinformatics methods based on population genetics should be further developed to quantitatively analyse the single-cell sequencing data. We developed a bioinformatics framework that was combined with molecular-evolution theories to analyse copy-number losses. This analysis revealed that most deletions in the breast cancers at the single-cell level were generated by simple stochastic processes. A non-standard type of coalescent theory, the multiple-merger coalescent model, aided by approximate Bayesian computation fit well with the data, allowing us to estimate the population-genetic parameters in addition to false-positive and false-negative rates. The estimated parameters suggest that the cancer cells underwent sweepstake evolution, where only one or very few parental cells produced a descendent cell population. We conclude that breast cancer cells successively substitute in a tumour mass, and the high reproduction of only a portion of cancer cells may confer high adaptability to this cancer.

## 1. Introduction

The idea that tumour progression can be viewed as a Darwinian process goes back to the 1970s, when it led to the concept of ‘clonal expansion’ [1]. During clonal expansion, tumour cells acquire rare advantageous mutations and then undergo rapid population expansion due to selection. This evolutionary process in tumours is strongly supported by recent genomic studies employing next-generation sequencing for a tumour mass, i.e. a mixture of tumour cells [2–7].

A more direct approach for evolutionary analysis is to study the genomes of individual cells. Single-nucleus sequencing (SNS) is a promising technology that generates high-resolution data, illustrating the genetic heterogeneity of cancer cells and providing an excellent tool for investigating the molecular evolution of cancer cells [8–13]. In SNS, single cells are isolated from tumour tissue by flow cytometry or micromanipulation, and then short DNA fragments (typically 50–200 bp) derived from a single cell are sequenced using a next-generation sequencer. The short sequenced reads are mapped to the human reference genome and then copy-number alterations (CNAs) or point mutations present in single tumour cells are identified by bioinformatics analysis. In particular, CNAs are detected based on the rationale that a larger number of reads mapping to a genomic region reflects a higher copy number in the region [8,14].

A recent study combined flow cytometry with next-generation sequencing and identified CNAs in the genomes of individual cells sampled from tumour tissues obtained from two patients with breast cancer [8]. Phylogenetic analysis of these CNA profiles revealed the existence of genetically distinct subpopulations, each of which was composed of homogeneous cells. These results suggested that breast cancer cells do not gradually evolve, but evolve rapidly between otherwise quiescent evolutionary periods. The results of other studies identified point mutations in the exomes of single cancer cells, and principal-component, phylogenetic-tree and allele-frequency analyses of these mutations revealed the mutational landscape of renal cell carcinoma and the monoclonal origin of essential thrombocythaemia [9,10].

Extensive efforts to develop analytical methods for cancer SNS data are mainly focused on the reconstruction of evolutionary trees such as phylogenetic trees (dendrograms in which the nodes represent cancer cells) [15,16] and mutation trees (dendrograms in which the nodes represent mutation sites) [17,18]. However, these methods assume data on point mutations, not CNAs. Tree reconstruction using SNS CNA data was introduced in a previous study [8], which employed the neighbour-joining method [19] based on the Euclidean distance between the integer copy numbers of cells. Although this distance shows some relatedness between cells, it is a population-genetic metric that may reflect correct genealogical relationships but is not well confirmed. Recently, a pipeline program to analyse SNS CNA data was developed [20]; however, this program focuses on quality control and CNA calling, and uses tree-reconstruction methods for which the distances have not been validated as appropriate metrics for phylogenetic-tree inference using copy numbers (e.g. the Euclidean distance between integer copy numbers). It is necessary to use a valid metric for reconstructing phylogenetic trees that reflects correct genealogies and further to develop an evolutionary model for understanding the dynamics of cancer cells that underlie the reconstructed trees.

For this purpose, we developed a population-genetic framework combined with bioinformatics techniques that analyses SNS CNA data, where cancer cells were treated as individuals of a non-sexually reproducing species. Based on this framework, we decoded integer copy numbers in the previous SNS CNA data [8] into genetic alleles, revealing an unexpectedly simple allelic nature for the breast cancers. We further found that individual cancer genomes fit well with an extended type of coalescent model, namely a multiple-merger coalescent (MMC) model [21] (reviewed in [22]) rather than the standard Kingman coalescent model, which is derived from the classic Wright–Fisher model [23]. MMC modelling allows multiple lineages to be merged simultaneously, based on a probability distribution for the number of merged lineages, whereas the Kingman coalescent model only allows the merger of two lineages. Our current findings explain why the phylogenetic tree showed distinct clades composed of homogeneous cells in a previous study [8], and suggest the underlying microscale dynamics of cancer cells in this cancer type.

## 2. Results

### 2.1. The nature of deletion alleles

Here, we analysed data generated in a previous study [8]. In the previous study, SNS was performed to identify integer copy numbers along binned chromosome regions (see the electronic supplementary material for more details regarding the data) for 100 single cells in a tissue designated as T10 and 100 single cells in tissues designated as T16P and T16M, which we collectively designate as T16 hereafter. Integer copy numbers ranged from 0 to 42, where ‘2’ represents the original diploid state, ‘0’ and ‘1’ represent deletions, and numbers greater than 2 indicate amplifications. The tissues were sampled from two patients diagnosed with ductal breast carcinoma. Tissue T10 was the primary carcinoma, and tissue T16 consisted of primary breast and metastatic liver carcinomas. Sampled cancer cells are considered as random samples because cells taken from the macro-dissected tissues were randomly selected and sorted by flow cytometry, and classified into ‘subpopulations’ with different ploidies based only on their DNA content, without any preference for particular cell types [8]. In this study, we focused only on the copy-number data and did not analyse somatic point mutations because there were few common sites among the cells, due to the low genomic coverage per cell (approx. 6% of the genome per cell).

**Figure 1.**
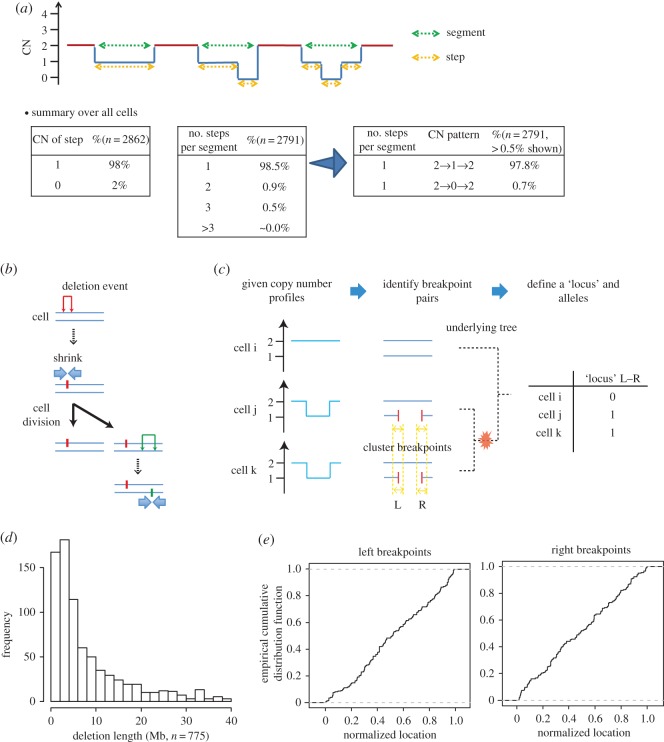
The nature of deletion alleles. Results from T10 only are shown because T16 showed essentially the same tendencies. (*a*) Profile patterns of the integer copy number (CN). The horizontal axis represents the chromosomal position. The general CN pattern ‘2→*n*→ … →*m*→2’ indicates that the copy numbers of a segment changed from 2 copies to *n* copies, … and to *m* copies finally back to 2 copies along a chromosome. (*b*) Evolutionary model of deletions. Every deletion event (the inverted U-shaped marks shown in red and green) leaves a unique pair of left and right breakpoints as fingerprints on a homologous chromosome (blue lines). (*c*) Procedure to convert copy-number profiles into alleles. ‘L’ and ‘R’ represent positions to the left and right of breakpoints, and an ‘L–R’ pair defines a locus. The symbols ‘0’ and ‘1’ represent the ancestral and derived alleles, respectively. (*d*) Distribution of deletion allele lengths. We excluded deletion alleles larger than the size of a chromosome level (40 Mb). (*e*) Distributions of breakpoint locations. The locations were normalized with respect to chromosome lengths.

First, we observed that the copy-number profiles were unexpectedly simple for cancer. The copy-number changes mostly involved 1-copy losses or gains (66% for T10 and 41% for T16), or, at most 2-copy losses or gains (87% for T10 and 76% for T16) (electronic supplementary material, figure S1*a*). In addition, the patterns along the binned chromosome regions were mostly simple, such as a pattern of 2 copies changing to 1 copy and back to 2 copies along the chromosome, from the start to end positions (electronic supplementary material, figure S1*a*). These simple copy-number patterns motivated us to perform a deeper analysis using population genetics. CNA segments were usually (91% for T10 and 96% for T16) composed of either amplifications or deletions, allowing us to analyse amplifications and deletions separately. Because the number of deletion states (0 or 1 copy) is lower than that of amplifications (3–42 copies), we focused on deletions to avoid theoretical complications in subsequent analyses. The deletion patterns were also simple (figure 1*a*).

Next, to perform population-genetics analysis, we considered the simple model of deletions illustrated in figure 1*b*, which assumes that any deletion event leaves a unique pair of right and left breakpoints on a chromosome. Under this assumption, all variable loci are bi-allelic, with original and derived allelic states. Because all chromosomes in a single cell are co-inherited by progeny cells, we treated them as if they evolved as a single chromosomal unit. Because paired homologous chromosomes are also inherited together, we assigned two different coordinate systems to a pair of homologous chromosomes, as if one homologous chromosome was physically linked to another by their ends.

Based on this model, we extracted deletion events as alleles. From the integer copy-number profiles for each cell, we obtained the left and right breakpoint pairs of the deletion events, using a simple greedy algorithm (figure 1*c*; electronic supplementary material for the details). We then performed clustering analysis to align the breakpoints across cells because breakpoints may fluctuate due to noise (figure 1*c*; electronic supplementary material). We defined a ‘genetic locus’ or ‘polymorphic site’ by a unique breakpoint pair and assigned a derived allele to cells that harboured the deletion. The ancestral allele was assigned to cells without the deletion. Results from principal-component analysis indicated that we successfully decoded the deletion alleles (electronic supplementary material, figure S1*b*).

We observed simple distributions for the deletion allele sizes and breakpoint positions. The number of deletion alleles gradually decreased according to size, which appeared to follow a reciprocal (1/*x*) distribution (figure 1*d*; electronic supplementary material, figure S1*c*). The breakpoints were distributed roughly uniformly across the genome (figure 1*e*). These data indicated that the deletion events occurred largely based on a relatively simple stochastic process, which was amenable to molecular-evolution analysis.

### 2.2. Multiple-merger coalescent

**Figure 2.**
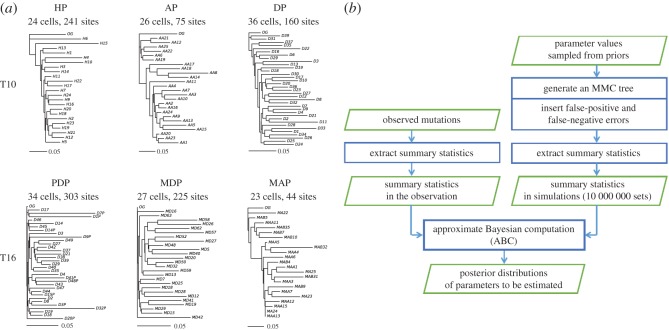

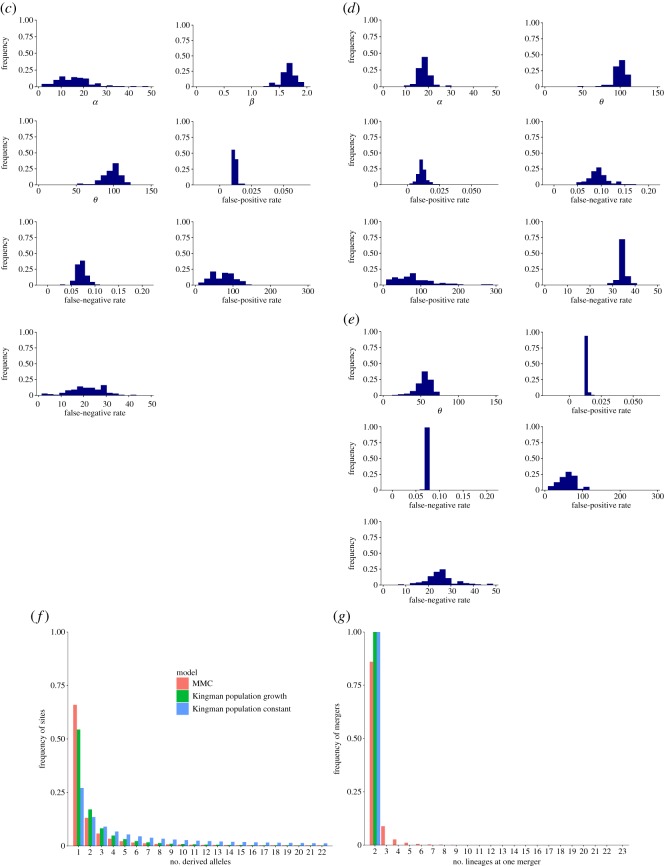
Phylogenetic trees and MMC. (*a*) Phylogenetic trees reconstructed by the neighbour-joining method. HP, AP, DP, PDP, MDP and MAP represent respective subpopulations of hypodiploid, aneuploid, diploid, primary diploid, metastatic diploid and metastatic aneuploid cells, which were defined previously [8]. OG represents the outgroup: no deletions at all sites. (*b*) Flow chart of our ABC. (*c*) The posterior distributions for the parameters of the MMC model. (*d*) The posterior distributions for the parameters of the Kingman population-growth model. (*e*) The posterior distributions for the parameters of the Kingman population-constant model. (*f*) Site-frequency spectrum under the MAP estimates. Sample size *n *= 23. (*g*) Distribution of the number of merged lineages under the MAP estimates. For (*f*,*g*), the results of 100 000 replications in the simulations were used.

We drew phylogenetic trees for the subpopulations defined previously [8]. Of all the subpopulations, the tree of the hypodiploid subpopulation (HP) had features expected from MMC theory: skewed branching and multiple mergers within a close distance (fig. 1 in [24]), as shown in figure 2*a* (by the neighbour-joining method) and in electronic supplementary material, figure S2 (by the unweighted pair group method with arithmetic mean [UPGMA]). The HP subpopulation most closely reflected the nature of deletions of all the subpopulations because HP was dominated by many copy-number losses [8]. Hence, we sought to analyse HP cells using an MMC model.

The objective of using an MMC model was to estimate various parameters that included a parameter related to the probability of multiple mergers. One of the simplest MMC models is the β-coalescent MMC model [22]. We used a modified β-coalescent model that included an exponential growth term (Material and methods section). Inclusion of the exponential growth term was justified by clinical observations with several types of cancer, including breast cancer [25]. We also modelled the occurrence of false-positive and false-negative errors in the data, because SNS data may have considerable errors.

Our model thus had seven parameters: the growth rate (*α*), the parameter (*β*) of the distribution that represents the rate of multiple mergers, population mutation rate (*θ*), false-positive and false-negative rates, and the numbers of false-positive and false-negative sites. The parameter α takes non-negative values, and a value of zero represents a constant population size. Values of *β* are defined within the range of 0–2. When *β* has a value close to 2, the rate distribution for the number (*m* in equation (4.1) in Material and methods section) of lineages to be merged has a large value for two lineages and smaller values for greater than two lineages. Indeed, the limit of 2 for *β* represents that only mergers of two lineages occur, as in Kingman coalescent models. When *β* has a value close to 0, it has larger values for greater than two lineages, meaning that multiple mergers (more than two lineages) tend to occur. Mutational events occur following a Poisson distribution with the mean of *θ *× *l*_b_ on a branch of a coalescent tree, where *l*_b_ is the branch length. False-positive sites are sites where alleles are originally copy-number neutral for all cells but are misjudged as deletions for some cells. False-negative sites are sites where alleles are originally deletion alleles for all cells but are misjudged as neutral for some cells.

We estimated these parameters in the framework of approximate Bayesian computation (ABC) (figure 2*b*) [26,27]. We used the features and summary statistics listed in table 1. The detailed reasons for selecting the features are described in electronic supplementary material, table S1. For comparison, we also used the models of Kingman coalescent with a constant population size and Kingman coalescent with a population growth. We obtained the posterior distributions (figure 2*c–e*) and maximum *a posteriori* probability (MAP) estimates (table 2). In the MMC model, the MAP result of *β* was 1.6, and the ratio of the posterior probabilities to the *β* value of nearly 2 (1.999) was 12.2. Hence, it appeared that HP cancer cells were better modelled by multiple mergers than by Kingman two-branch mergers.

**Table 1.** Features and summary statistics. The reasons for selecting these features are listed in electronic supplementary material, table S1.

feature	summary statistics
number of mutation sites	the number itself
allele frequencies at all sites	10, 30, 50, 70 and 90% quantiles
distances between all cell pairs in a tree	10, 30, 50, 70 and 90% quantiles
all branch lengths in a tree	10, 30, 50, 70 and 90% quantiles
associations (*r*^2^) between all site pairs	10, 30, 50, 70 and 90% quantiles

**Table 2.** MAP estimates. *α* and *β* represent the population-growth rate and the parameter of the distribution that describes the rate of multiple mergers, respectively. See the text for more information on *α* and *β*. Here, *θ* is the population mutation rate.

parameter	the MMC model	Kingman population-constant model	Kingman population-growth model
*α*	17.1	n.a.	17.7
*β*	1.64	n.a.	n.a.
*θ*	102.9	52.1	97.2
false-positive rate	0.012	0.013	0.010
false-negative rate	0.068	0.072	0.098
false-positive sites	89	72	70
false-negative sites	21	26	35

Formally, we calculated the posterior probabilities of the three models in model selection when we used the multinomial logistic regression with explanatory variables for the summary statistics. The posterior probabilities were 1.00, 0.00 and 0.00 for the MMC, Kingman population-constant and Kingman population-growth models, respectively, which suggests that MMC was the best model. We confirmed that it was possible to distinguish the three models when we used the multinomial logistic regression by performing a leave-one-out cross-validation analysis of the misclassification rates of the models (misclassification rate of only 1.7% on average; electronic supplementary material, table S2).

In addition, we performed two analyses that complement the analysis of models' posterior probabilities [28]. We first performed the goodness-of-fit test using, as the test statistics, the distance between the accepted summary statistics and observed summary statistics for each model. The *p*-values were 0.37, 0.18 and 0.05 for the MMC, Kingman population-constant and Kingman population-growth models, respectively. This suggests that the Kingman population-growth model was significantly deviated from observed data, and that the MMC model was the best fit among the three.

Second, we performed posterior predictive checks for each model, where we checked the concordance between summary statistics calculated from observed data and summary statistics calculated from coalescent simulations performed secondarily, based on 1000 sets of parameter values sampled from the initially obtained posterior distributions for the parameters (electronic supplementary material, figure S3). In addition, we checked the concordance between the observed summary statistics and summary statistics secondarily simulated under the MAP estimates (electronic supplementary material, figure S3). These results showed that the summary statistics were reasonably reproduced for all of the models, although the Kingman population growth and constant models were least concordant in the predictive checks based on the posteriors and MAP estimates, respectively. In summary, the MMC model was always the best model across all the three analyses of model selection.

We also calculated an allele-frequency spectrum (figure 2*f*). This spectrum was drawn based on the estimated parameters without false-positive or false-negative errors, because a spectrum (electronic supplementary material, figure S4) constructed directly from the observed data might have been contaminated by false positives and negatives. For comparison purposes, we calculated spectra for Kingman population-constant and population-growth models. The *β* coalescent with growth showed an intense frequency at the smallest number of derived alleles and sharp drops in the frequencies at large numbers, particularly at the second smallest number (figure 2*f*). The Kingman growth model showed a less intense frequency at the smallest number, but did not show as sharp a drop in the frequency at the second smallest number. The Kingman constant model did not have an intense frequency at the smallest number and had long-tail frequencies at large numbers. We also computed a distribution for the number of merged lineages observed during the simulation. Mergers of more than two lineages were found (figure 2*g*). Both Kingman models were two-lineage mergers by definition.

## 3. Discussion

In this study, we developed a computational framework that integrates bioinformatics copy-number algorithms with population-genetics theory. Using this approach, we quantitatively analysed the previous SNS CNA data in breast cancers [8]. Our analyses of copy-number profiles and deletion alleles demonstrated that their patterns were unexpectedly simple for cancer. Other investigators proposed the fractal globule model to explain the 1/*x* distribution of CNA sizes in typical bulk-cell sequencing [29,30], and our analysis demonstrated that this observation held true at the single-cell level. The 1/*x* distribution, together with the uniform distribution of breakpoint positions in chromosomes, may serve as a future simulation framework for modelling stochastic processes of CNAs in cancer cells. Lengths around branch mergers in the HP tree (figure 2*a*) mostly appeared short enough to be approximated with multiple mergers; indeed, MMC fit better to the CNA data than it did to the Kingman coalescent models.

A phylogenetic analysis by Navin *et al*. [8] demonstrated the presence of distinct subpopulations composed of homogeneous cancer cells; no clear intermediate subpopulations were found in the breast cancer cells they examined. The absence of intermediate subpopulations can be explained by ‘sweepstake’ reproductive processes underlying the MMC model. Unlike the Wright–Fisher model or the Kingman coalescent model, MMC is characterized by great variance in the number of descendants: the MMC models are coalescent processes in species with ‘sweepstake’ reproduction such as fish and parasites, in which only one or very few individuals produce descendants [21,22,31]. The population is composed of very few genotypes. Therefore, cancer cells within the same subpopulation were genetically *homogeneous* in the previous study [8]. On the other hand, the time of allele fixation in the sweepstake reproduction modelled in MMC is short; hence, many divergences (substitutions) tend to accumulate between two incipient populations [22]. This is the reason why *distinct* subpopulations were observed in the previous study [8].

One important prediction by MMC is that alleles under positive selection theoretically may have a probability of 1 to become fixed [22]. The possibility that even a slightly advantageous allele can be fixed under a little genetic drift may be related to numerous ‘passenger’ mutations observed in recent cancer-genomics studies [2,32].

There are several biological and medical implications if the cancer data fit the MMC model.

(1) To understand how cancer is generated in a human body, cancer genomics employing typical next-generation sequencing for bulk cells estimates the order of dysfunctional genes from the variant allele frequencies in a tumour tissue sample, based on the idea that older variants have higher variant allele frequencies [33,34]. For example, if variants in *KRAS* and *TP53* show variant allele frequencies of 50% and 30%, it is estimated that the *KRAS* variant occurred before the *TP53* variant. In this example, *KRAS* is interpreted as a possible initiating factor for this cancer. This reconstruction holds true in the Wright–Fisher and Kingman models; however, it is not true in the MMC model because higher variant frequencies may just reflect variants occurring in a rapidly expanding subpopulation [35]. If the data fit the MMC model well, this order reconstruction method may be incorrect.

(2) As with the management of marine species [31], the reproductive skew in the MMC model has implications for the management of cancer treatment. The reproductive skew is represented by a heavy-tailed *Cx*^−^*^β^* distribution for the probability of having *x* or more offspring, where *C* is a positive constant [31,36]. If data from a cancer patient fit with the MMC model, it suggests that killing cancer cells randomly with anti-cancer drugs would be ineffective because the surviving cells with very high reproduction located in the heavy tail of the distribution will surely re-emerge. It is much more effective to distinguish such cells using biomarkers and kill them directly. The cancer stem cell hypothesis suggests that only a small portion of cancer cells with stem cell properties generate mitotic descendent cells, which constitute almost all of the cancer cell population [37]. This hypothesis may be associated with the high reproductive skew represented in the MMC model, and some markers (e.g. CD44^+^/CD24^−^ for breast cancer) to distinguish cancer stem cells have already been developed.

(3) If the cancer data fit the MMC, it is disadvantageous to take a wait-and-see approach because even slightly advantaged variants may spread through the population and cancer cells rapidly evolve; thus, an estimated *β* can serve as an index to represent the malignancy of the cancer.

To our knowledge, this is the first study to apply MMC modelling to cancer SNS data. Branching processes have been often used to model cancer evolution [13,38]. Branching processes are a time-forward type of model, while MMC is a time-backward type of model. The standard Kingman model as a backward model can be derived from the Wright–Fisher model as a forward model [23]. In this light, it is interesting that recent theoretical studies indicated a relationship between MMC as a backward model and branching processes as a forward model [39,40].

Although multiple deletion events may share the same breakpoint pairs, we ascertained that virtually all deletions in the dataset arose from single events (see the electronic supplementary material). One caveat in our analysis is that we only examined simple deletions defined from copy-number profiles. We did not identify deletions within amplifications, let alone amplifications or point mutations. Future studies are warranted to include these mutations. Moreover, if data with a sufficient number of cells were obtained from every dissected sector of a tissue, ‘geographic’ differences in tissues could be addressed in the future. The mutational model of CNAs depicted in figure 1*b* and also suggested in a previous study [41] is in principle applicable to germ-line copy-number variations (CNVs) and therefore may also be helpful for improving population-genetic studies of CNVs [42,43].

## 4. Material and methods

### 4.1. Phylogenetic tree

Because we extracted genetic alleles from copy-number profiles, we applied standard phylogenetic-construction methods that are used for point mutations. For the distance, we used the *p*-distance [44] because multiple occurrences were unlikely to occur, as described in the electronic supplementary material. We used the neighbour-joining method [19] for agglomeration, in addition to the UPGMA method. We constructed trees for subpopulations with greater than 20 cells.

### 4.2. Multiple-merger coalescent

In our coalescent simulation, we used a β-coalescent model modified to include population growth. We based our simulation procedures on reference [24]. In the β-coalescent model [22], the rate of merger of *m* lineages from *k* active lineages is represented by

4.1 $$\begin{array}{rl} \lambda_{k,m} & =\int_{0}^{1}x^{m-2}{\left(1-x\right)}^{k-m}\left\{\frac{1}{\Gamma (2-\beta )\Gamma (\beta )}x^{1-\beta }{\left(1-x\right)}^{\beta -1}\right\}\;\text{d}x\\ & =\frac{B(m-\beta ,k-m+\beta )}{B(2-\beta ,\beta )}, \end{array}\}$$

where *Γ* and *B* represent the gamma and beta functions, respectively, and *β* is the parameter. The number of lineages to be merged is first sampled from the probabilities:

4.2 $$p_{k,m}=\left(\begin{array}{c} k\\ m \end{array}\right)\lambda_{k,m}.$$

Next, particular lineages to be merged are randomly sampled. This process is repeated until no lineages remain.

Coalescent time *t_k_* under the assumption of a constant population size is simulated in [24] as follows:

4.3 $$t_{k}∼\text{Exponential}(p_{k})$$

and

4.4 $$p_{k}=\sum_{m=2}^{k}p_{k,m},$$

where *t_k_* is sampled from an exponential distribution with a rate of *p_k_*. Intuitively, coalescent events occur, following a Poisson process with the average number of occurrences of *p_k_* in coalescent time units.

We scaled the waiting times to include the effect of population growth, following the standard approach in coalescent theories [45]. This approach focuses only on changes in the coalescent rate due to changes in population size, i.e. smaller population size is associated with a higher coalescent rate, and scales the coalescent time appropriately. We assumed an exponential growth model:

4.5 $$N(t)=N_{0}{\text{e}}^{-\alpha t},$$

where *N*_0_ is the population size at the present and *t* is the time before the present; *α* is the growth rate measured in coalescent time units (4*N*_0_ generations). Exponential growth can be included in a coalescent model by scaling time as follows:

4.6 $$t_{k}^{*}∼\text{Exponential}(p_{k}),$$

where $t_{k}^{*}$ is sampled from an exponential distribution with a rate of $p_{k}$. The time is then scaled with the growth rate *α*:

4.7 $$t_{k}=\frac{1}{\alpha }\log(1+\alpha t_{k}^{*}{\text{e}}^{-\alpha v_{k+1}}),$$

and

4.8 $$v_{k+1}=\sum_{i=k+1}^{n}t_{i},$$

where *n* is the sample size. We thus obtained the coalescent tree and time.

For each branch of an MMC tree, we sampled the number of mutational events from a Poisson distribution with the mean of *θ *× *l*_b_, where *θ* is the population mutation rate (in coalescent time units) and *l*_b_ is the branch length. We then placed mutational events onto the branch. As shown in figure 1*c*, we treated the breakpoint pair of a deletion as a point mutation that follows the infinite-site model [46].

### 4.3. Approximate Bayesian computation

We first sampled values from prior distributions, assuming uniform distributions:

*α*: {0, 10, 20, 30, 40, 50, 60, 70, 80, 90, 100},
*β*: {0.001, 0.2, 0.4, 0.6, 0.8, 1.0, 1.2, 1.4, 1.6, 1.8, 1.999},
*θ*: {10, 20, 30, 40, 50, 60, 70, 80, 90, 100},
False-positive rate: {0.01, 0.02, 0.03, 0.04, 0.05, 0.06, 0.07, 0.08, 0.09, 0.1},
False-negative rate: {0.02, 0.04, 0.06, 0.08, 0.10, 0.12, 0.14, 0.16, 0.18, 0.2},
Number of false-positive sites: {10, 20, 30, 40, 50, 60, 70, 80, 90, 100},
Number of false-negative sites: {10, 20, 30, 40, 50, 60, 70, 80, 90, 100}.

Here, the false-positive sites are sites that had copy-number-neutral alleles for all cells originally, but were misjudged to have a deletion allele for at least one cell. Conversely, false-negative sites are sites that had deletion alleles for all cells originally, but were misjudged to have a neutral allele for at least one cell.

Under a set of parameter values, we generated an MMC tree with mutations. From the tree, we obtained DNA sequences where derived and ancestral alleles were represented as ‘1’ and ‘0’, respectively. We then simulated false positives and negatives by performing Bernoulli's trials with the probability of the given false-negative and false-positive rates, and then flipped the alleles (‘1’ to ‘0’ or ‘0’ to ‘1’) based on the outcomes of the trials, respectively. The same method was applied to Bernoulli's trials for single sites where the alleles were all ‘1's (or ‘0's) across the cells. It follows that we obtained sites where at least one ‘1’ (or ‘0’) was flipped over. Then, we added such sites to the DNA sequence data up to the given number of false-negative (or false-positive) sites. In this way, we simulated DNA sequences with false positives and false negatives.

We then extracted five features and their summary statistics, as given in table 1. The reasons for selecting these features are described in electronic supplementary material, table S1. The reason for using the summary statistics of quantiles is that we wished to use information as close to the distribution itself as possible. We repeated these processes 10 000 000 times to obtain 10 000 000 sets of summary statistics.

Using the ‘abc’ package [47] of R, we compared the summary statistics obtained with the simulated data with those with the observed data, based on ABC with the ridge regression adjustment (method=‘ridge’ in the ‘abc’ function) [26]. We determined the acceptance rate to be 0.001%, based on prediction errors calculated from 100 cross-validations for each parameter at different acceptance rates by the ‘cv4abc’ function (electronic supplementary material, table S3). We used features of the tree reconstructed from the neighbour-joining method for the ABC features related to a tree.

For the population-growth Kingman model, we fixed *α* to 0. For the population-constant Kingman model, we further fixed *β* to 1.999 (approx. 2). For these models, we performed the same ABC procedures that were performed for the *β* coalescent with growth.

In model selection analysis, we used the ‘postpr’ function to calculate the posterior probabilities of the three models in the multinomial logistic regression, and used the ‘cv4postpr’ function to perform a leave-one-out cross-validation analysis for the misclassification rates of the three models. We used the ‘gfit’ function to perform a leave-one-out cross-validation for the goodness-of-fit test using a statistic of the distance between the accepted summary statistics and the observed summary statistics.

## Supplementary Material

All Supplementary Materials

## Data accessibility

The datasets supporting this article have been uploaded as part of the electronic supplementary material. The source codes for MMC simulations are freely available from Dryad (http://dx.doi.org/10.5061/dryad.71jp0) [48].
