# Supplementary material for: Sweepstake evolution revealed by population-genetic analysis of copy-number alterations in single genomes of breast cancer
Source: R Soc Open Sci. 2017 Sep 27;4(9):171060. doi: 10.1098/rsos.171060 (PMC5627131; doi:10.1098/rsos.171060)

Supplementary Text

Supplementary Methods

Copy-number profile data

Integer copy numbers in the genome for each cell were obtained from data reported by Navin *et al.* [1], using single-nucleus sequencing technology. In their study, the integer copy numbers were defined based on the principle that a larger number of NGS reads mapped to the bins of chromosomes reflected a higher integer copy number. Specifically, simulations were performed to generate NGS reads by randomly sampling 200 million genomic sequences with a length of 48 bp from the chromosomes of the human reference genome. Then, they mapped the simulated NGS reads to the reference genome using a mapping tool. They defined chromosomal bins that were expected to have the same number of mapped sequence reads (the bin sizes were variable and the median size was 53 kb). They next counted the number of mapped sequence reads in real NGS data in each bin for each cell, and measured the deviation of the count from the expected count to assign integer copy numbers to each bin for each cell. They finally connected neighbouring bins to form CNA segments such that single segments were composed of only consecutive bins with non-2 copy numbers.

Copy number profiles to alleles

To determine deletion alleles from copy-number profile data, we made the following 2 assumptions. (1) No total copy numbers over homologous chromosomes were counter-balanced by a combination of amplifications and deletions (*e.g.*, a total of 2 copies may be counter-balanced by a 1-copy gain in one homologous chromosome and a 1-copy loss in the other, at the same location). (2) Every deletion event causes a 1-copy loss and this mutational change does not revert.

Using integer copy numbers assigned to the bins that divided chromosomes, we identified the left and right breakpoints of deletion events for each cell by a simple greedy algorithm. Specifically, we represented the left and right breakpoint pair of a

deletion event by bin indices (in integers): [L, $L + 1$; R, $R + 1$], where the left breakpoint exists between L and $L + 1$, and the right breakpoint exists between R and $R + 1$. Here, the bin indices are integers that uniquely specify bins, and are serially numbered from left to right along a chromosome.

The algorithm first defines the breakpoint pair of a deletion event by the leftmost and rightmost breakpoint pair of a deletion segment. Then, the algorithm artificially increases the copy numbers within the segment by one copy and repeats this process to obtain further breakpoint pairs. Supplementary Methods Figure 1 illustrates an example of the algorithm. Consider a scenario where the copy numbers are [2, 1, 0, 2] at bin indices [1, 2, 3, 4]. The algorithm first obtains an event with the breakpoint pair represented by bin indices, [1, 2; 3, 4], and then it artificially increases the copy numbers to [2, 2, 1, 2]. Finally, it obtains another event with breakpoint pair [2, 3; 3, 4]. The implicit assumption of this simple algorithm is that, when it is theoretically possible to decompose a segment in multiple ways, the algorithm should select the way that involves an event with the shortest length. For example, consider a case with copy numbers [2, 1, 0, 1, 2] at bin indices [1, 2, 3, 4, 5]. These data can be decomposed into events with a breakpoint pair [1, 2; 4, 5] and with [2, 3; 3, 4], or events with [1, 2; 3, 4] and with [2, 3; 4, 5]. The algorithm chooses the former. Nevertheless, because copy-number patterns with multiple possibilities were limited (1.5% for T10 and 0.3% for T16), this assumption was rarely applied.

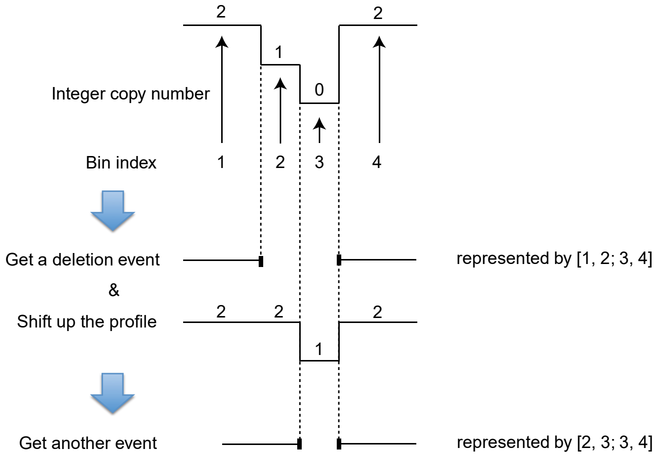

Supplementary Methods Figure 1. Illustration depicting how to obtain the breakpoints of deletion events from integer copy numbers.

After we obtained breakpoints for each cell, we performed a clustering analysis to align the breakpoints across cells, considering that the breakpoint positions may fluctuate due to noise. We separately clustered the Ls and Rs sites by hierarchal clustering, while retaining information regarding cell IDs and paired breakpoints. For

example, we retained information that L_1 was paired with R_1 and that it belonged to cells 1, 2, and 10 in the clustering of *L*s. Such information was used to restrict agglomeration during clustering, as described below, and to restore paired breakpoints after clustering. Performing hierarchal clustering requires 2 parameters: the distance between 2 clusters and an agglomeration method. For distance, we used the absolute difference between the medians of the bin indices of clusters. For the agglomeration method, we selected the cluster pair with the smallest distance from all possible cluster pairs. We restricted possible cluster pairs to those of which the cell IDs were mutually exclusive because we assumed that the original breakpoint shifted due to noise in some cells; consequently, we did not agglomerate breakpoints arising from the same cell. When multiple cluster pairs demonstrated the minimum distance, we chose one pair at random (we confirmed by means of several trials that this random choice did not markedly affect the results). The agglomeration process was repeated until the minimum distance crossed a threshold value. For a set of clustered *L*s (*R*s), we used the rounded median as the representative bin index.

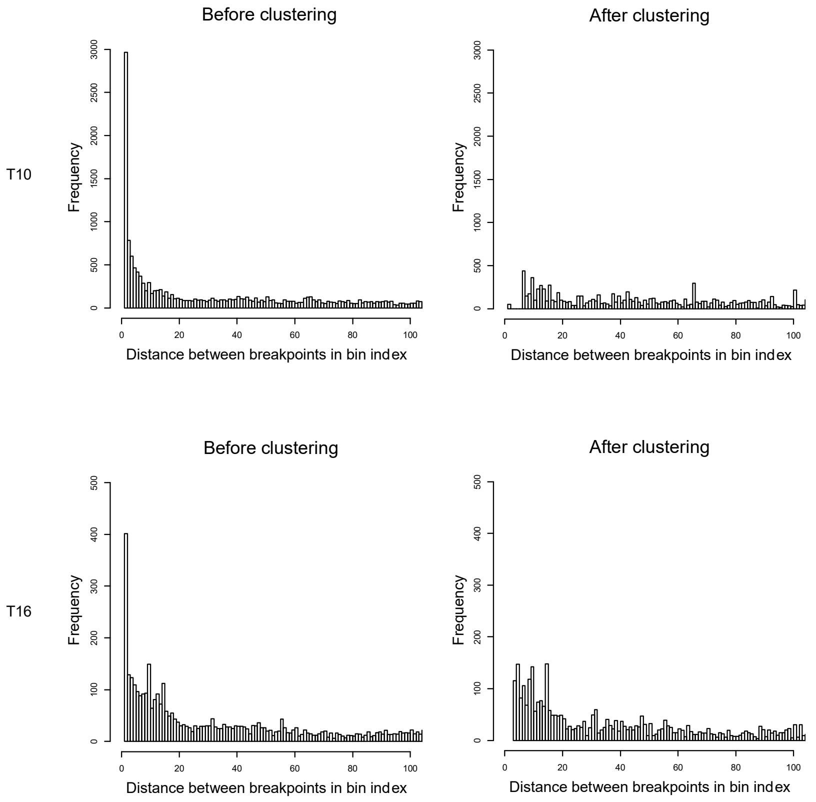

Supplementary Methods Figure 2. The effect of clustering on the distances between breakpoints. The distances between *L*s were calculated separately from those between *R*s.

The effect of this clustering is demonstrated in Supplementary Methods Figure 2. The *x*-axis represents the absolute value of the

difference in bin indices between every pair of bins having breakpoints. For example, if bins with breakpoints are expressed by bin indices 1, 3, and 4, then we select the bin-index pairs 1 and 3, 1 and 4, and 3 and 4 and hence the absolute values of the differences are 2, 3, and 1, respectively. Before clustering, the distribution of the distance values showed a vast number of occurrences at a 1-bin difference and a large number of occurrences at differences of a few bins. This result indicated that the breakpoints frequently shifted by one bin or, less frequently, by a few bins due to noise. We tested 10 thresholds (1–10) for the minimum distance and opted for the smallest threshold (6 for T10 and 3 for T16) that gave a distribution that diminished these extraordinary peaks at short distances.

Next, we defined alleles from these breakpoint pairs. That is, when cells shared the same L and R values, we inferred that the breakpoint pair was inherited from a mutational event that occurred in an ancestral cell at some point in the evolutionary history. We regarded the breakpoint pair as a fingerprint of the mutational event. Therefore, we defined the pair as the derived “allele” at “locus” [L, $L + 1$; R, $R + 1$]. No distinction was made between 2 homologous chromosomes; therefore, this definition included an implicit parsimonious assumption: when cells shared the same breakpoint pair, we assumed that 1 mutational event occurred on 1 homologous chromosome during the coalescence. Cells that did not show deletion fingerprints at a locus were considered to harbour ancestral alleles. The bin indices of the loci were converted into chromosomal positions in the hg18 coordinates.

Supplementary Results

Evaluation of multiple hits

To confirm that we did not underestimate the number of deletions due to multiple hits, we calculated the probability of multiple deletion events sharing the same breakpoint pair in chromosomal bins that were used to detect CNAs. Motivated by observations of deletion lengths and breakpoint locations in our data, we used the model in which 1 end of a breakpoint pair occurs uniformly along a chromosome, and the tract length is determined by the fractal globule model [2, 3]; that is, the length follows a reciprocal distribution. As shown in Supplementary Figure S1, our data suggested that the

reciprocal distribution approximated an exponential distribution within the length range. Because it is easier to estimate the parameters for exponential distributions, we approximated the reciprocal distribution using an exponential distribution. This approximation appeared to be valid for a discussion of orders of magnitude in probability values.

Because the number of initial breakpoints per base or even per copy-number bin is considered to be small, this number can be assumed to follow a Poisson distribution. Then, the probability of 1 deletion defined with a breakpoint pair $\boldsymbol{x} = [L_l, L_r; R_l, R_r]$ is:

$$P_1 = Po(1|\hat{\lambda}) \cdot \{E_L(\boldsymbol{x}|\hat{l}) + E_R(\boldsymbol{x}|\hat{l})\} \quad (1)$$

where L_l and L_r represent the chromosomal positions of the left and the right bins for the left breakpoint, and R_l and R_r represent the corresponding values for the right breakpoint. $Po(k|\hat{\lambda})$ represents a Poisson distribution of k occurrences with $\hat{\lambda}$, the average occurrences per bin (and the time interval from the most recent common ancestor to the current cell). $E_L(\boldsymbol{x}|\hat{l})$ and $E_R(\boldsymbol{x}|\hat{l})$ represent the probability that the tract extends to the left and the right directions, respectively. This probability is calculated as follows:

$$E(\boldsymbol{x}|\hat{l}) = \frac{1}{2d}\int_0^d \int_{x+a}^{x+b} e(l|\hat{l})\, dldx = \frac{\hat{l}}{2d}\left[e^{-\frac{x+b}{\hat{l}}} - e^{-\frac{x+a}{\hat{l}}}\right]_0^d \quad (2)$$

where $e(l|\hat{l})$ is an exponential distribution of length l with the average length of $\hat{l}$. x is the distance from the initial breakpoint within $[L_l, L_r]$ (or $[R_l, R_r]$) to L_r (R_l), and a and b are the distances from L_r to R_l (R_l to L_r) and from L_r to R_r (R_l to L_l), respectively. d is the distance from L_l to L_r (R_l to R_r). The inner integral represents the cumulative probability of the exponential distribution from $x + a$ to $x + b$. The outer integral represents the average of the probabilities over x moving $[0, d]$. The factor of 1/2 corresponds to the tract extending in 1 direction.

The probability that 2 deletions occur with the same breakpoint pair $\boldsymbol{x}$ is:

$$P_2 = Po(2|\hat{\lambda}) \cdot \left\{E_L(\boldsymbol{x}|\hat{l})^2 + E_R(\boldsymbol{x}|\hat{l})^2\right\} + Po(1|\hat{\lambda})E_L(\boldsymbol{x}|\hat{l}) \cdot Po(1|\hat{\lambda})E_R(\boldsymbol{x}|\hat{l}) \quad (3)$$

where the left term represents the initial breakpoints of 2 deletions occurring within the same bin and with their tracts extending in the same direction, and the right term indicates that 1 initial breakpoint is present within 1 bin and its tract extends in 1 direction, while the other initial breakpoint is present within another bin and its tract

extends in the opposite direction. We can evaluate the relative probability of 2 hits to 1 hit using the formula $P_2/(P_1 + P_2)$. The probability of more than 2 hits occurring is much lower, by orders of magnitude, than the probability of only 2 hits occurring; hence, we ignored the former probability here.

We estimated $\hat{\lambda}$ as a Poisson-corrected distance (as defined for point mutations), assuming a uniformity of $\hat{\lambda}$ across the bins. That is,

$$Po(0|\hat{\lambda}) = e^{-\hat{\lambda}} = 1 - (N_+/2)/N \Leftrightarrow \hat{\lambda} = -\log(1 - (N_+/2)/N) \quad (4)$$

where $N_+/2$ is half the observed number of bins with any breakpoints (an initial breakpoint is either on the left or on the right) and N is the number of all bins. The assumption of this uniformity appears valid at an average resolution of 1 Mb or more because the coefficient of variation of the number of breakpoints was only 34% (for a mean of 0.056) in approximately 1-Mb successive bins (a series of 20 copy-number bins, *i.e.*, 20 × ca. 50 kb = ca. 1 Mb). Note that the sizes of the copy-number bins were varied, but in essence remained virtually unchanged (as 97% of them ranged from 46 to 69 kb, with a median of 54 kb); hence, the uniformity of $\hat{\lambda}$ was not significantly affected by the bin size. For $\hat{l}$, we simply calculated the mean length over deletions.

Based on this model, a probability of 2 hits sharing the same breakpoint pair relative to that of a single hit was found to be 2×10^{-6} (based on the HP data, which had the most deletions among all the subpopulations). The expected number of events over the genome was found to be 8×10^{-4}. Thus, all deletions appear to have come from single hits.

Supplementary Tables

Supplementary Table S1. Reasons for selecting the ABC features.

Feature	Reason for selecting this feature
Number of mutation sites	This feature is directly linked to, and is important for determining, the population mutation rate, θ. Note that in the ABC procedure, the number, taking into account false positives/negatives in the simulation, was compared with the observed number of mutation sites.
Allele frequencies at all sites	The most fundamental quantity in population genetics. Information on mutation rates, selective force, and demographic factors such as population growth is summarized by this feature.
Distances between all cell pairs in a tree	The largest difference in a tree between MMC and the standard coalescent is reflected by this feature [4], where 1 peak appears near 0 in the distribution in MMC, while multiple peaks appear in the standard coalescent. This feature does not depend on the reconstruction methods of a tree.
All branch lengths in a tree	Coalescent processes are determined by 2 parameters: coalescent time and the number of nodes merged. The specific definitions of the 2 parameters separate the Kingman models (with and without growth) from MMC, and their outcomes are reflected in this feature. This feature depends on the reconstruction methods of a tree.
Associations (r^2) between all site pairs	Observation errors are expected to give r^2 of 0; alleles constrained by phylogeny are expected to give non-0 r^2 values in many cases.

Supplementary Table S2. Misclassification rates of the models.

Real/predicted	MMC	Kingman population-constant	Kingman population-growth	Misclassification rate
**MMC**	99	0	1	0.010
**Kingman population-constant**	0	98	2	0.020
**Kingman population-growth**	2	0	98	0.020

Supplementary Table S3. The prediction errors of the parameters.

Model	Tolerance	α	β	θ	False positive rate	False negative rate	# of false positive sites	# of false negative sites
MMC	1.00E-06	0.914	0.77	0.881	0.162	0.204	1.176	0.083
	1.00E-05	*0.814*	*0.585*	*0.851*	*0.166*	*0.261*	*0.915*	*0.147*
	1.00E-04	0.937	0.742	0.92	0.18	0.415	1.319	0.123
Kingman population-constant	1.00E-06	NA	NA	0.245	0.201	0.164	1.163	0.348
	1.00E-05	*NA*	*NA*	*0.24*	*0.145*	*0.188*	*1.124*	*0.347*
	1.00E-04	NA	NA	0.248	0.199	0.23	1.332	0.419
Kingman population-growth	1.00E-06	0.761	NA	0.912	0.189	0.142	0.583	0.077
	1.00E-05	*0.74*	*NA*	*0.876*	*0.198*	*0.154*	*0.518*	*0.062*
	1.00E-04	0.888	NA	0.893	0.224	0.175	0.529	0.092

The prediction error is calculated as

$$E = \frac{\sum_i (\tilde{\theta}_i - \theta_i)^2}{Var(\theta_i)} \quad ,$$

where θ_i is the true parameter value of the *i*-th simulated data set in a leave-one-out cross-validation and $\tilde{\theta}_i$ is the estimated parameter value. A tolerance of 10^{-5} was finally used in our ABC because it resulted in the smallest errors.

References

1 Navin, N., Kendall, J., Troge, J., Andrews, P., Rodgers, L., McIndoo, J., Cook, K., Stepansky, A., Levy, D., Esposito, D.*, et al.* 2011 Tumour evolution inferred by single-cell sequencing. *Nature*. **472**, 90-94. (doi:10.1038/nature09807)

2 Fudenberg, G., Getz, G., Meyerson, M., Mirny, L. A. 2011 High order chromatin architecture shapes the landscape of chromosomal alterations in cancer. *Nature biotechnology*. **29**, 1109-1113. (doi:10.1038/nbt.2049)

3 Mirny, L. A. 2011 The fractal globule as a model of chromatin architecture in the cell. *Chromosome research : an international journal on the molecular, supramolecular and evolutionary aspects of chromosome biology*. **19**, 37-51. (doi:10.1007/s10577-010-9177-0)

4 Tellier, A., Lemaire, C. 2014 Coalescence 2.0: a multiple branching of recent theoretical developments and their applications. *Molecular ecology*. **23**, 2637-2652. (doi:10.1111/mec.12755)

Supplementary Figure Legends

Supplementary Figure S1. The nature of CNAs supplement to figure 1.
(*a*) Copy-number profiles, including gene amplifications. The horizontal axis represents chromosomal position. (*b*) Principal component analysis (PCA) based on the decoded deletion alleles. We removed outlier cells (8 DP cells, 1 HP cell, and 3 PAP cells) from the PCA plot and from subsequent analyses. As expected from a previous study [1], we observed a clear separation among the DP, HP, and AP subpopulations, and between the PDP/MDP and PAP/MAP subpopulations in the plot. It was also anticipated that we could not distinguish PDP from MDP (or PAP from MAP) in T16. (*c*) Cumulative distribution function (CDF) of deletion lengths. We generated random values, using the mean of the observed data for the exponential distribution and their minimum and maximum values for the reciprocal distribution.

Supplementary Figure S2. UPGMA trees.
Phylogenetic trees reconstructed by the UPGMA method

Supplementary Figure S3. Predictive checks.
(*a*) Posterior predictive checks. (*b*) Predictive checks based on the MAP estimates.

Supplementary Figure S4. The allele frequency spectrum constructed directly from the observed data.

Supplementary Figure S1

(*a*)

• Deletions and amplifications

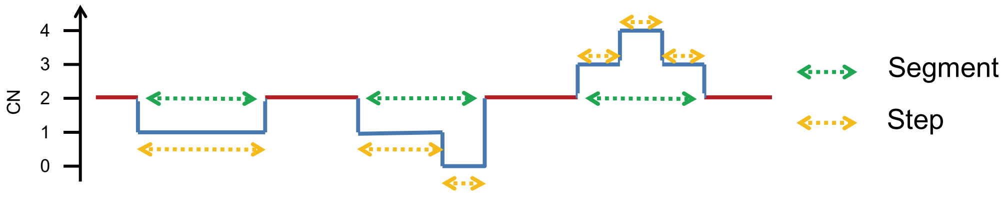

• Summary over all cells

CN of step	% (n = 7584)
0	1%
1	37%
3	29%
4	18%
>4	15%

# of steps per segment	% (n = 4553)
1	75%
2	12%
3	4%
4	2%
>4	7%

# of steps per segment	CN pattern	% (n = 4553 >2% shown)
1	2->1->2	50%
1	2->3->2	16%
1	2->4->2	5%
2	2->4->3->2	3%
2	2->3->1->2	2%
2	2->1->3->2	2%

(*b*)

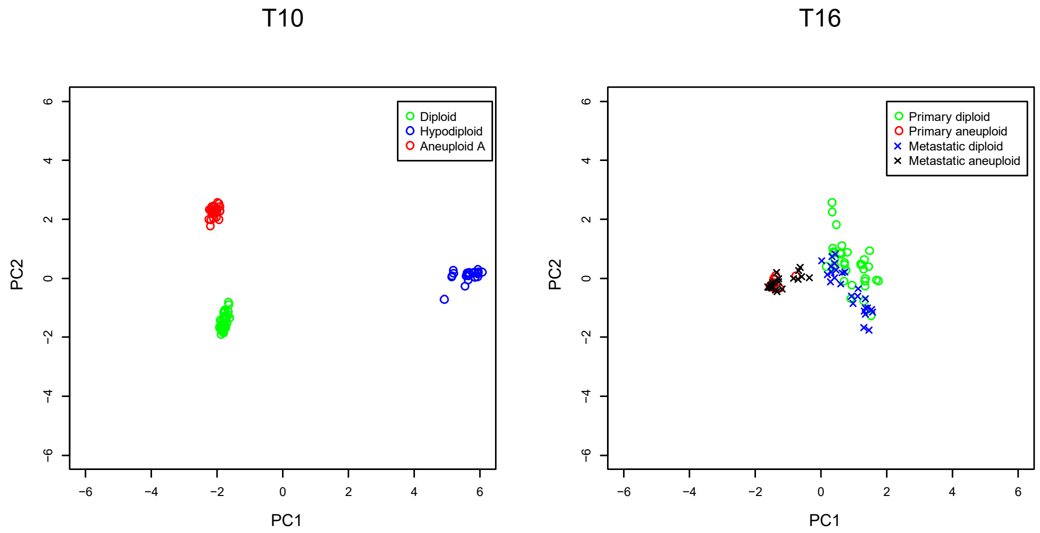

(*c*)

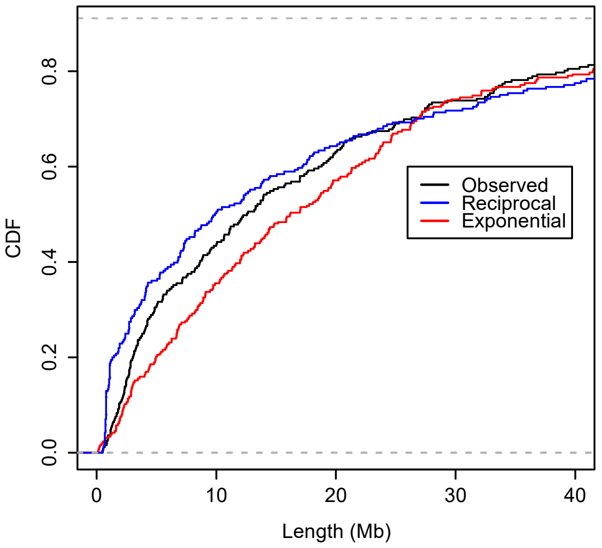

Supplementary Figure S2

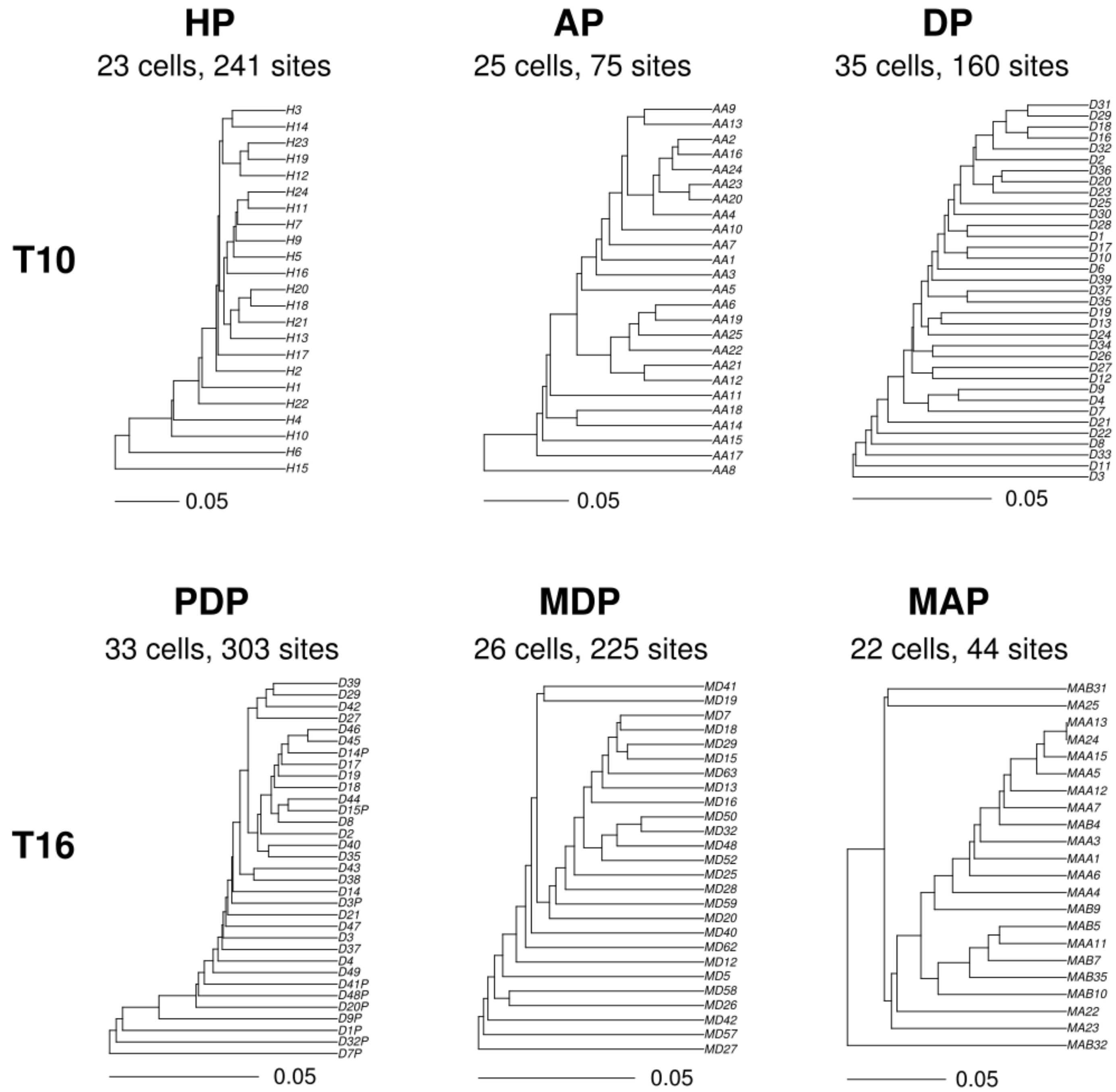

Supplementary Figure S3

(*a*)

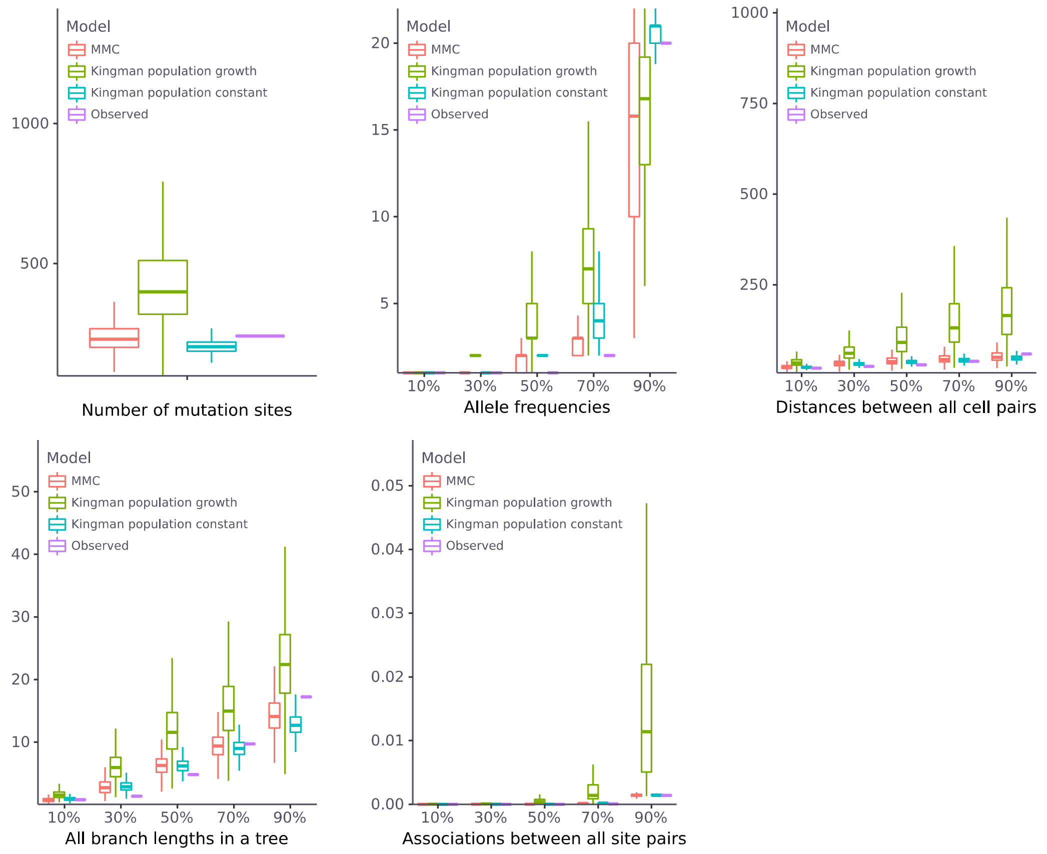

(*b*)

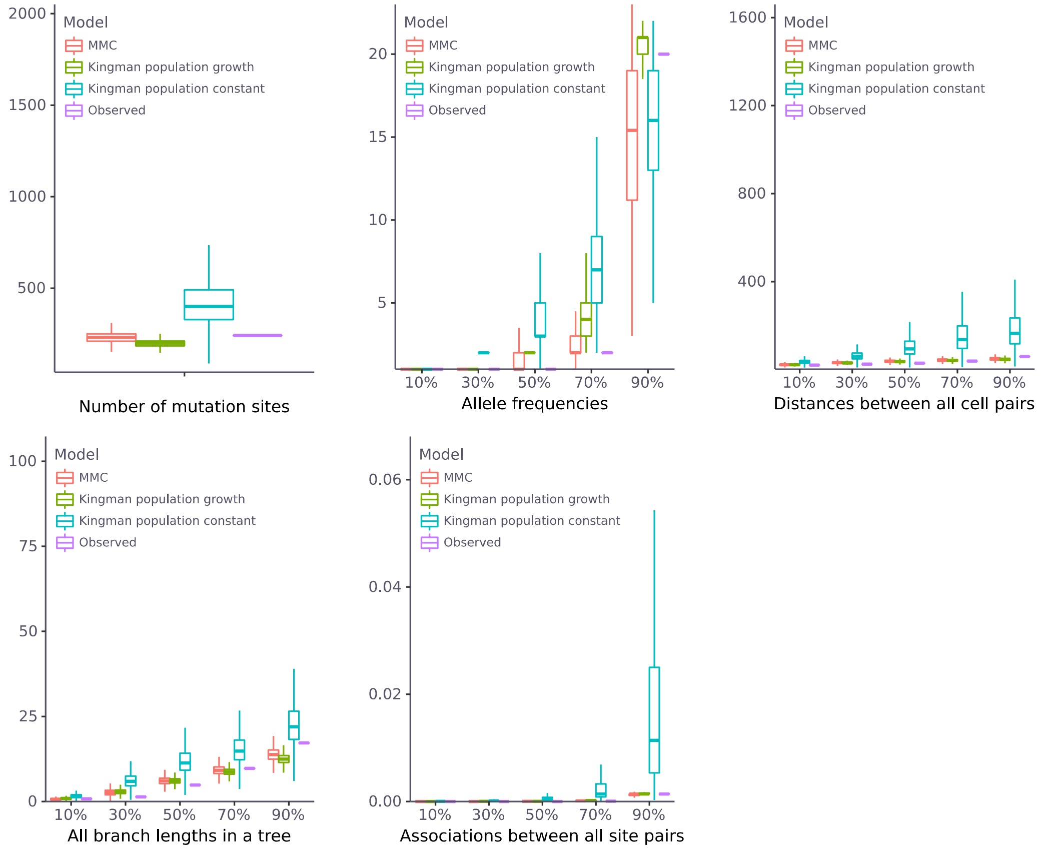

Supplementary Figure S4

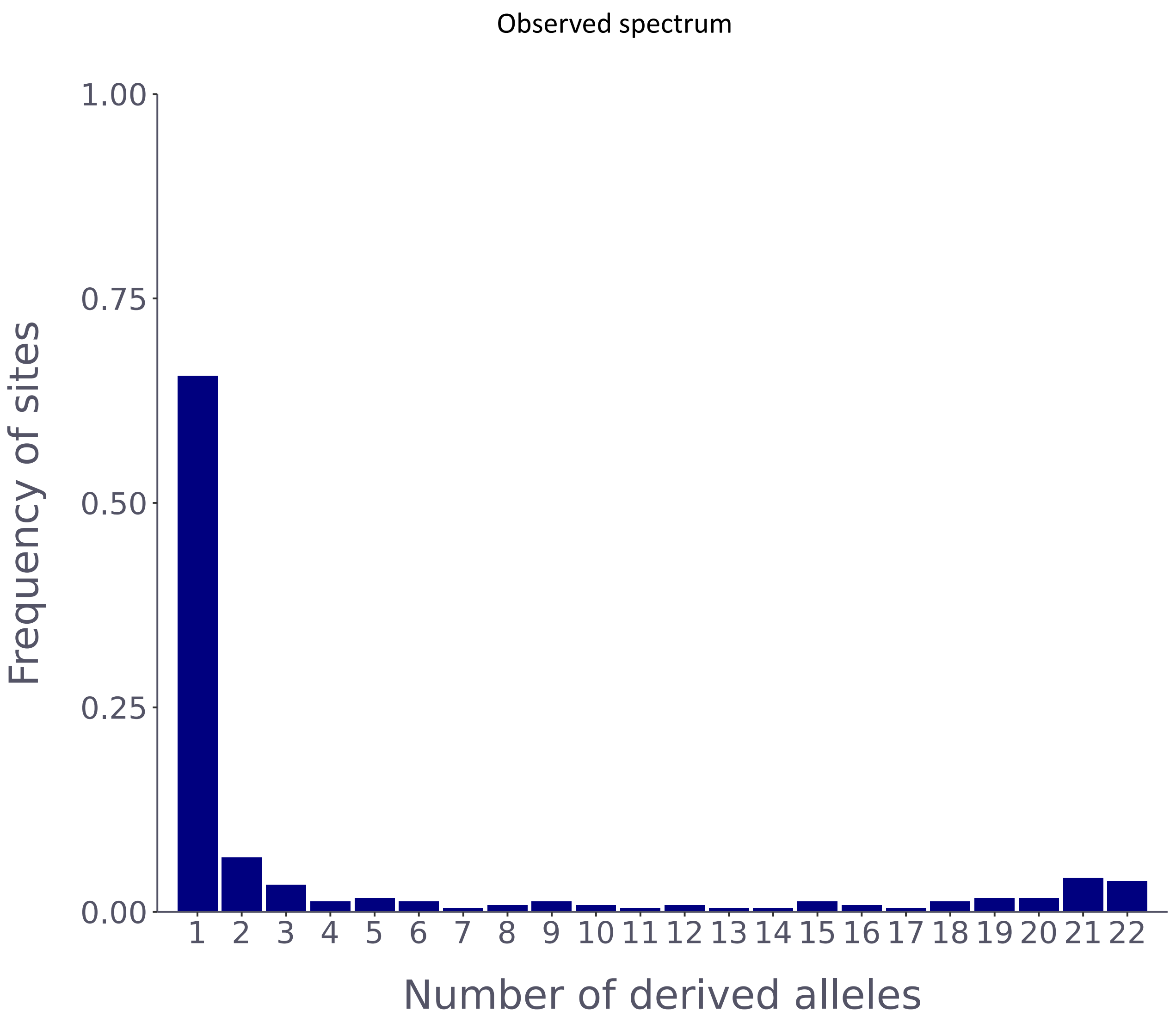

Supplement: All Supplementary Materials [file rsos171060supp1.pdf]
